# Semaglutide treatment attenuates vessel remodelling in ApoE−/− mice following vascular injury and blood flow perturbation

**DOI:** 10.1016/j.athplu.2022.05.004

**Published:** 2022-06-04

**Authors:** Ditte Marie Jensen, Gry Freja Skovsted, Mathilde Frederikke Bjørn Bonde, Jacob Fog Bentzon, Bidda Rolin, Grégory Franck, Maria Katarina Elm Ougaard, Louise Marie Voetmann, Julian Christoffer Bachmann, Anna Uryga, Charles Pyke, Rikke Kaae Kirk, Henning Hvid, Lotte Bjerre Knudsen, Jens Lykkesfeldt, Michael Nyberg

**Affiliations:** a Department of Veterinary and Animal Sciences, University of Copenhagen, Ridebanevej 9, 1870 Frederiksberg C, Copenhagen, Denmark; b Research and Early Development, Novo Nordisk A/S, Novo Nordisk Park 1, 2760, Maaloev, Denmark; c Department of Clinical Medicine, Heart Diseases and Steno Diabetes Center Aarhus, Palle Juul-Jensens Boulevard 99, Aarhus University, 8200, Aarhus, Denmark; d Centro Nacional de Investigaciones Cardiovasculares (CNIC), Alle Melchor Fernandez Almagro, 3, 28029, Madrid, Spain; e INSERM U1148, Laboratory for Vascular Translational Science, Bichat Hospital, 46 Rue Henri Huchard, Paris, France

**Keywords:** Atherosclerosis, Glucagon-like peptide 1 receptor agonists, Semaglutide, Vascular smooth muscle cells, Vascular injury, Phenotypic switching, Plaque erosion, *ApoE*^−/−^, Apolipoprotein E knock-out, GLP 1RA, Glucagon-like peptide 1 receptor agonist, IHC, Immunohistochemistry, ISH, In situ hybridisation, LCCA, Left common carotid artery, SSP1, Osteopontin, SoC, Standard of care, VSMC, Vascular smooth muscle cells

## Abstract

**Background and aims:**

Randomized clinical studies have shown a reduction in cardiovascular outcomes with glucagon-like peptide 1 receptor agonist (GLP-1RA) treatment with the hypothesized mechanisms being an underlying effect on atherosclerosis. Here, we aimed to assess the pharmacological effects of semaglutide in an atheroprone murine model that recapitulates central mechanisms related to vascular smooth muscle cell (VSMC) phenotypic switching and endothelial dysfunction known to operate within the atherosclerotic plaque.

**Methods:**

In study A, we employed an electrical current to the carotid artery in ApoE−/− mice to induce severe VSMC injury and death, after which the arteries were allowed to heal for 4 weeks. In study B, a constrictive cuff was added for 6 h at the site of the healed segment to induce a disturbance in blood flow.

**Results:**

Compared to vehicle, semaglutide treatment reduced the intimal and medial area by ∼66% (p = 0.007) and ∼11% (*p* = 0.0002), respectively. Following cuff placement, expression of the pro-inflammatory marker osteopontin and macrophage marker Mac-2 was reduced (*p* < 0.05) in the semaglutide-treated group compared to vehicle. GLP-1R were not expressed in murine carotid artery and human coronary vessels with and without atherosclerotic plaques, and semaglutide treatment did not affect proliferation of cultured primary human VSMCs.

**Conclusions:**

Semaglutide treatment reduced vessel remodelling following electrical injury and blood flow perturbation in an atheroprone mouse model. This effect appears to be driven by anti-inflammatory and -proliferative mechanisms independent of GLP-1 receptor-mediated signalling in the resident vascular cells. This mechanism of action may be important for cardiovascular protection.

## Highlights

• We assessed the pharmacological effects of semaglutide in atheroprone mice.
• The model recapitulates central mechanisms related to VSMC phenotypic switching.
• Semaglutide treatment reduced VSMC proliferation and vessel remodelling.
• This effect was independent of GLP-1 receptor-mediated signalling in vascular cells.
• Such mechanism of action may be important for the cardiovascular protection.

## 1 Introduction

The increasingly widespread use of effective pharmacological lipid-lowering and antihypertensive agents as standard of care (SoC) has played an essential role in reducing the burden of atherosclerotic disease [1]. Nevertheless, the residual risk of an atherosclerotic event remains considerable. This may be related to the failure of current therapies to effectively counteract cell-specific mechanisms operating locally within the plaque lesion. Vascular smooth muscle cells (VSMCs) and VSMC-derived cells comprise a major fraction of cells present at all stages of an atherosclerotic plaque [2], and combined genetic and functional experimental data have also provided evidence for a causal role of phenotypically switched VSMCs in atherosclerotic plaque development and progression [[3], [4], [5], [6]].

Cardiovascular outcome trials with glucagon-like peptide-1 receptor agonists (GLP-1RA) have shown a significantly reduced number of major cardiovascular events in high-risk patients with diabetes already receiving SoC [[7], [8], [9], [10]]. In the LEADER trial, a significant treatment effect in the subgroup of patients with established cardiovascular disease was observed, suggesting an effect of GLP-1RAs on atherosclerosis [11] that is mediated via mechanisms distinct from those targeted by SoC. Along these lines, treatment of VSMCs *in vitro* with GLP-1RAs has been proposed to modulate the expression of matrix metalloproteinases and inhibit VSMC proliferation and migration [12,13] and treatment with liraglutide reduced intima-media ratio in an atheroprone murine model [14], indicating that GLP-1RAs may influence VSMC phenotypic switching. However, more evidence is needed to support such a mode of action.

While effective pharmacological lowering of lipids and blood pressure has reduced the incidence of clinical events caused by rupture of lipid-rich and inflamed plaques, the incidence of plaque erosion, caused by thrombus formation on lipid-poor and non-inflamed plaques after superficial shedding of the endothelium, have remained high [1]. Eroded atheromas are abundant in VSMCs, collagen, glycosaminoglycans, and proteoglycans [15], and it has been suggested that alterations in structure and function in the subendothelial layer are needed for superficial erosion to occur [16]. Hence, GLP-1RAs may modulate VSMC phenotypic switching and consequently the development of plaque erosion. However, experimental evidence for this mechanism is lacking.

Recently, a murine model system that features key components of human plaque erosion was presented [17]. This model employs electric injury of the carotid artery that then heals for 4 weeks with resulting formation of VSMC- and matrix–rich neointima and regenerated endothelial cells. Subsequently, the healed segment is exposed to disturbed blood flow by applying a constrictive perivascular cuff which leads to denudation of the endothelial layer as observed in plaque erosion. Notably, the phenotypic switching, proliferation, and migration of VSMCs, which drive the remodelling of the vessel wall following electric injury in the murine model has also been described in human atherosclerotic plaque formation and progression [2,18]. Therefore, this experimental approach/setup also provides a model system for elucidation of specific VSMC-related mechanisms relevant to atherosclerosis.

Accordingly, the present study aimed to test the treatment effects of the GLP-1RA semaglutide on injury-induced vessel remodelling and the local response to acute disturbances in blood flow at the previously injured site in atheroprone ApoE−/− mice. We hypothesized that semaglutide would attenuate vessel remodelling as evidenced by decreased neointima formation following electric injury and reduce desquamation of endothelial cells as a result of cuff-induced alterations in blood flow.

## 2 Materials and methods

An expanded Materials and methods section is available in the Supplementary Material.

### 2.1 Animals

Male apolipoprotein E knock-out (*ApoE* ^−/−^) mice (total n = 155) (B6.129P2-Apoetm1Unc/J), six to nine weeks old (Jackson Laboratories, USA, stock 002052, and Charles River, Italy) were randomly assigned to groups upon arrival. Animals were housed solitarily and allowed an acclimatisation period for at least two weeks before entering the study. The mice had unlimited access to tap water and standard chow (Altromin 1324, Brogaarden, Denmark) and were housed in an enriched environment with standard bedding and nesting material, under a 12/12 h day-night cycle in a humidity (30–70%) and temperature (20–25 °C) controlled facility. All animal procedures followed the guidelines for the care and handling of laboratory animals established by the Danish government. The Danish Animal Experimental Inspectorate under the Danish Ministry of Food, Fisheries and Agriculture and the Novo Nordisk Animal Welfare Body approved the studies (permission 2015-15-0201-00688). All animals were inspected daily by animal caretakers.

### 2.2 Experimental design

Three animal studies were designed; an electric injury study (Study A), a plaque erosion study (Study B), and a constrictive cuff study (Control study). Supplementary Fig. 1 shows the timeline and details of the animal studies conducted. Group sizes in study A and B were chosen to be able to detect a difference of >20% in neointimal area between vehicle and semaglutide treated animals. In the Control study, the group size reflects the number of animals needed to detect qualitative differences with acute placement of the constrictive cuff.

#### Study A: electric injury study

This study aimed to investigate the effects of semaglutide dosing on vascular injury done by electric injury on the left carotid artery (LCCA). Animals (n = 71) were euthanized 28 days after electric injury. Animals were administered daily semaglutide (n = 31) or vehicle (n = 30) during the study period. Semaglutide and vehicle-dosed animals received electric injury surgery, whereas control animals (n = 10) were sham operated.

#### Study B: plaque erosion study

This study aimed to investigate the effects of semaglutide dosing on vascular injury done by electric injury on the LCCA and subsequently induction of turbulent flow on the injured area by applying a perivascular constrictive cuff 28 days after electric injury. Animals (n = 77) were administered daily semaglutide (n = 32) or vehicle (n = 36) during the study period. Semaglutide and vehicle-dosed animals received electric injury surgery, whereas control animals (n = 9) were sham operated. Animals were euthanized 6 h after cuff application.

Control study: Constrictive cuff. This study aimed to investigate the effects of a perivascular constrictive cuff per se on vascular remodelling after 6 h. A perivascular constrictive cuff was applied on the LCCA as done in Study B, but without the electric injury step. Animals (n = 7) were euthanized 6 h after the cuff was applied.

### 2.13 Statistical analysis

Data was analysed using the software GraphPad Prism version 9.0.1 (GraphPad Software Inc., La Jolla, CA, USA). The assumptions of normally distributed data and variance homogeneity was examined by visual inspection of qq-plots and residual plots, respectively. If data deviated from a normal distribution, and/or displayed variance heterogeneity, statistical analysis was performed on log-transformed data or with the use of non-parametric test. Within each experiment, differences in means between groups for each parameter were analysed using either a one-way ANOVA or Kruskal-Wallis test with Tukey's post hoc test. Furthermore, to obtain a general estimate of the effect of treatment in study A and B for the parameters intima thickness, media thickness, Mac-2 area and osteopontin area, log-transformed data from these two studies were combined and analysed in a linear model with the factors treatment (three levels: vehicle, semaglutide or control) and experiment (two levels: Study A or Study B) and the possible interaction between these two factors included, using the software SAS JMP version 14 (SAS Institute, Cary, NC, USA) as done in Ref. [19]. No significant interaction between treatment and study was found and with the linear models, the mean differences between treatment groups and corresponding 95% confidence intervals were estimated on a log-scale with Tukey's adjustment for multiple comparisons. The mean differences and limits of the corresponding 95% confidence intervals were finally back-transformed to obtain ratios. On all graphs data are presented as mean ± SEM, and in all analyses *P-*values <0.05 were considered statistically significant.

## 3 Results

### 3.1 Semaglutide reduced bodyweight and plasma lipid/cholesterol in ApoE^−/−^ mice

**Fig. 1:**
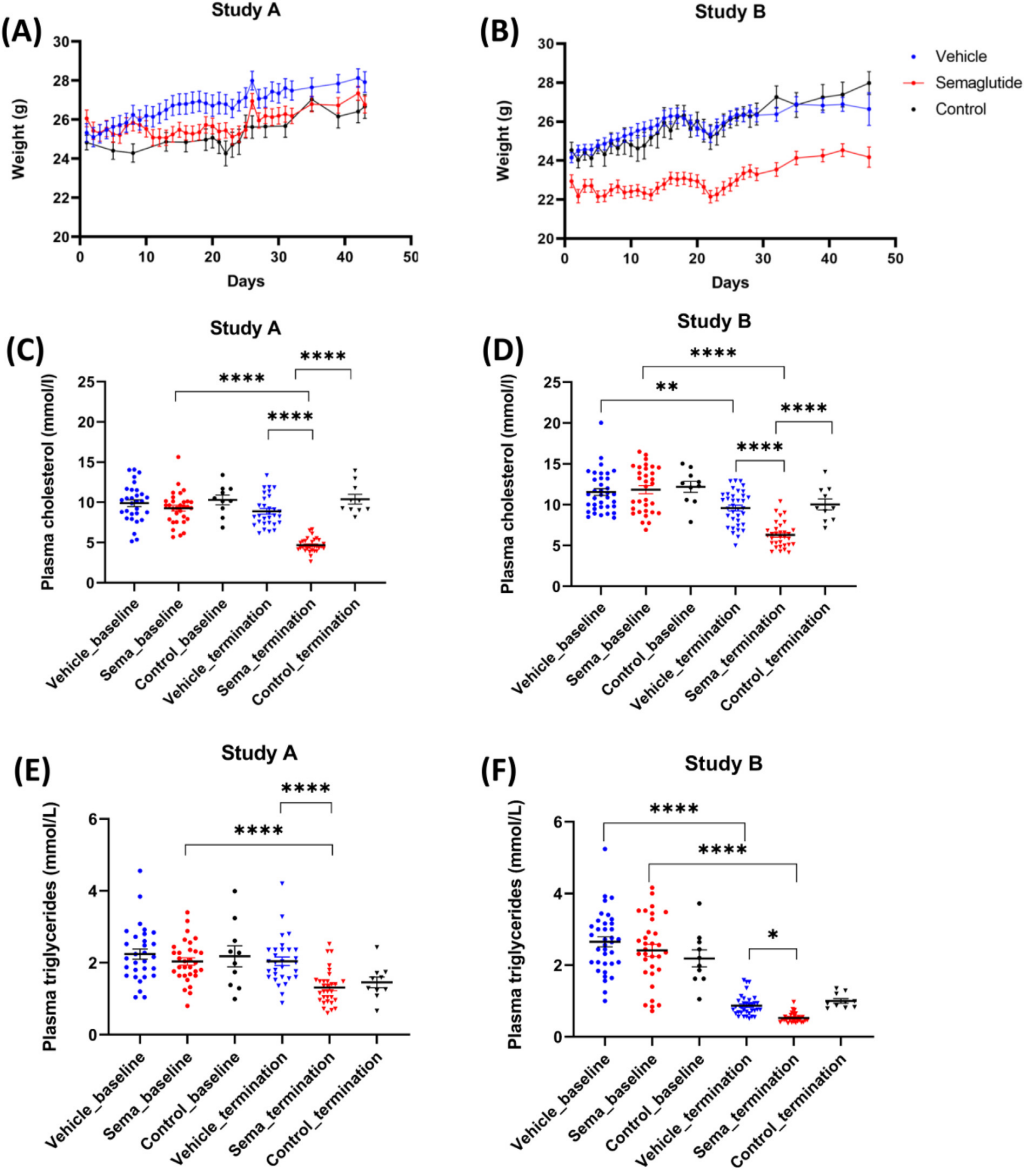
Body weight and lipid parameters. (A) Body weight at study start (baseline, day 0) for mice in Study A (electric injury) and Study B (plaque erosion)(B). Each data point is the average of animals in that group on the specific day. (C) Plasma cholesterol (mmol/L) at baseline and termination in mice from Study A (electric injury) and B (plaque erosion)(D). (E) Plasma triglyceride (mmol/L) at baseline and termination in mice from Study A (electric injury) and B (plaque erosion) (F). Statistical significance:∗*p* < 0.05, ∗∗*p* < 0.01, ∗∗∗*p* < 0.001, ∗∗∗∗*p* < 0.0001. Results are shown as mean ± SEM.

Details on weight and blood parameters (cholesterol and triglycerides) at baseline and termination are given in Fig. 1 and show that groups were comparable at study start (day 0) for all parameters. In both studies, semaglutide administration resulted in a decrease in body weight compared with vehicle treatment. Semaglutide had a significant effect on plasma total cholesterol levels in both studies, reducing levels by 34% (p < 0.0001) and 21% (p < 0.0001), respectively, at termination compared to vehicle. Plasma triglyceride was significantly reduced compared to vehicle at termination in study A (2.04 ± 0.12 mM in vehicle vs. 1.31 ± 0.09 mM in semaglutide p < 0.0001) and study B (0.87 ± 0.05 mM in vehicle vs 0.53 ± 0.02 mM in semaglutide, p < 0.05).

### 3.2 Electric injury resulted in neointima formation and inflammation in the LCCA vessel wall

**Fig. 2:**
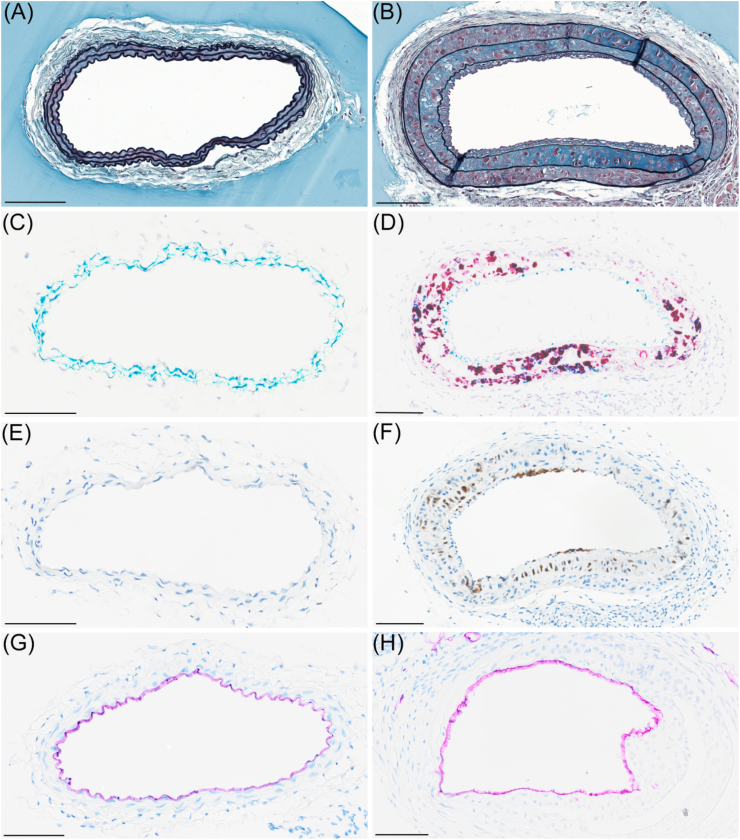
Histological depiction of vessels with (right panel) and without neointima (left panel). (A) Carotid artery without neointima (Movat's pentachrome stain). (B) Carotid artery with neointima showing increased tunica media and tunica intima area (Movat's pentachrome stain). (C) Carotid artery without neointima stained for Myh11 (teal) and osteopontin (red) (duplex ISH). (D) Carotid artery with neointima stained for Myh11 (teal) and osteopontin (red) (duplex ISH). (E) Carotid artery without neointima stained for Mac-2 (IHC). (F) Injured carotid artery with neointima stained for Mac-2 (IHC). (G) Carotid artery without plaque stained for CD31 (IHC). (H) Carotid artery with injury stained for CD31 (IHC). Scalebar is 100 μm in length.

Representative images of histological sections of LCCA vessels with or without neointima formation are shown in Fig. 2. Vessels with neointima were characterised by marked expansion of the tunica intima (the neointima) and tunica media (Fig. 2A and B). Normal vessels expressed Myh11 in the tunica media and not osteopontin (Fig. 2C), whereas osteopontin expression in vessels with neointima increased markedly in the tunica media (Fig. 2D). Likewise, Mac-2 (a macrophage marker), was either absent or expressed at very low levels in normal vessels (Fig. 2E), but expressed at relatively high levels in neointima areas (Fig. 2F). Normal vessels as well as the areas with neointima were covered by intact endothelium, visualized by IHC for CD31 (Fig. 2G and H). Electric injury and electric injury combined with a constrictive cuff for 6 h resulted in qualitative comparable staining patterns. Supplementary Fig. 2 outlines the histological sampling of LCCA. Quantification of tunica intima and tunica media is depicted in Supplementary Fig. 3, and Supplementary Fig. 4 depicts the quantification of IHC and ISH stained sections.

### 3.3 Semaglutide reduced the marked expansion of tunica intima and media induced by electric injury

**Fig. 3:**
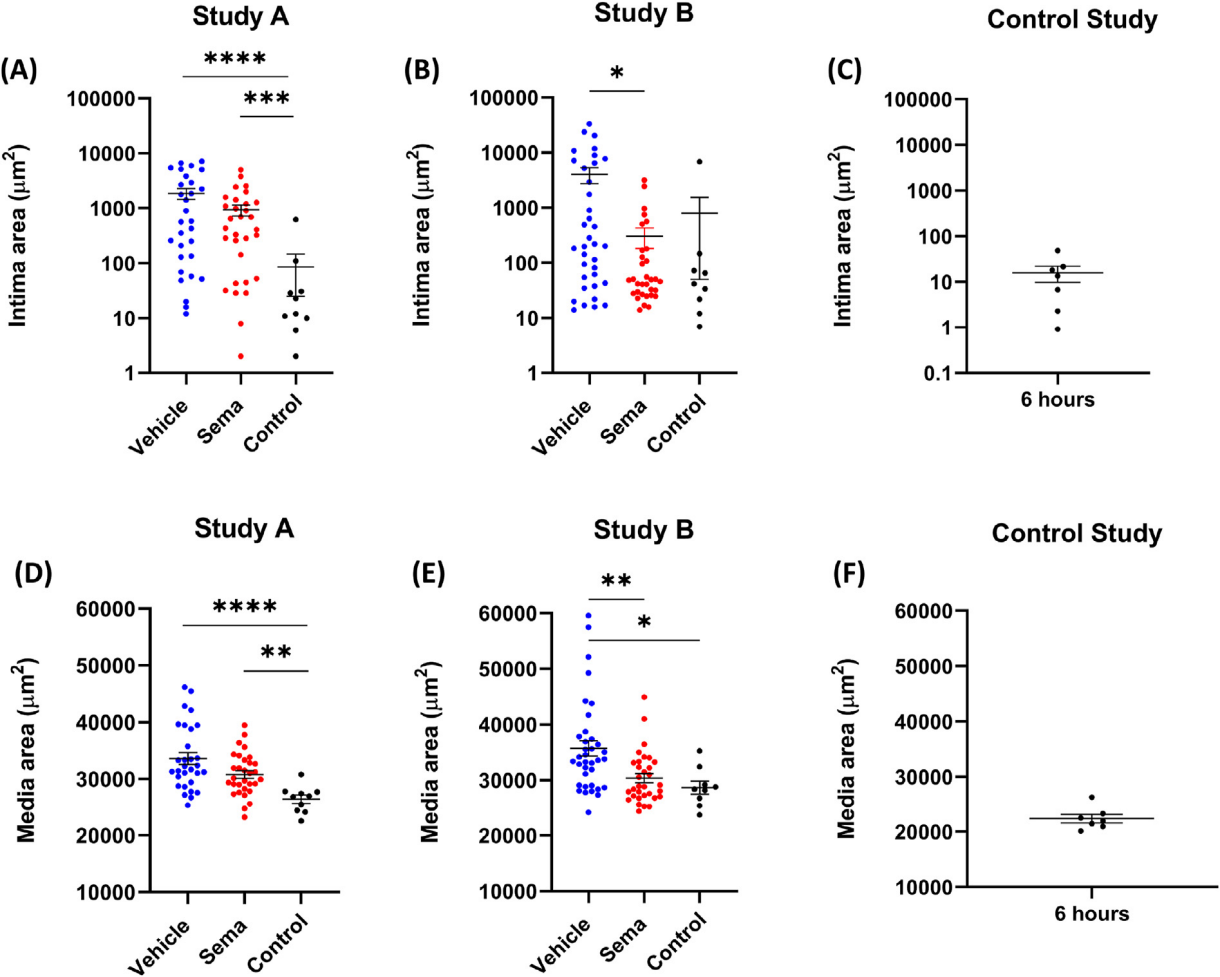
Area of tunica intima and tunica media. (A) Intima area in mice from Study A (electric injury). (B) Intima area in mice from Study B (plaque erosion study). (C) Intima area in mice from the control study (constrictive cuff). (D) Tunica media area in mice from Study A (electric injury). (E) Tunica media area in mice from Study B (plaque erosion). (F) Tunica media area in mice from the control study (constrictive cuff). Intima and media area in Study A and Study B were quantified from approximately 40 slides in total per animal (every 10th pentachrome-stained slide starting from the bifurcation end). In the control study, intima and media area was quantified from approximately 20 slides in total per animal (every 10th pentachrome-stained slide starting from where the end of the cuff). Each data point represents one animal. Statistical significance:∗p < 0.05, ∗∗p < 0.01, ∗∗∗p < 0.001, ∗∗∗∗p < 0.0001. Results are shown as mean ± SEM. Note that the y-axis is log scale in (A), (B) and (C).

As shown on Fig. 2, electric injury resulted in marked expansion of the tunica intima and media. When quantified with image analysis, the tunica intima area was significantly higher in vehicle-treated mice compared to control in study A (*p* < 0.0001, Fig. 3A). Tunica intima area was also elevated in vehicle-treated mice compared to control in study B, where electric injury was combined with application of a constrictive cuff for 6 h, but it did not reach statistical significance (Fig. 3B). Application of a cuff alone for 6 h did not influence the area of the tunica intima (Fig. 3C). In mice treated with semaglutide, the intima area was increased compared to the control-treated mice in study A (*p* = 0.0006, Fig. 3A). In a combined analysis of study A and B, electric injury resulted in 12.5-fold increase of intima area (*p* < 0.0001) compared to control group, and this was reduced by 66% in the semaglutide treated mice (*p* = 0.007), see Table 1. In Supplementary Figs. 5–7, the details of tunica intima area versus depth (slide number) are visualized for individual animals in Study A, B and the control study.

**Table 1:** Combined analysis of Study A and Study B.

Parameter	Comparison	Ratio	95% CI lower limit	95% CI upper limit	*p*-value
Intima thickness	Vehicle vs. Control	12.7	3.7	43.3	<0.0001
	Semaglutide vs. Vehicle	0.34	0.15	0.78	0.007
Media thickness	Vehicle vs. Control	1.24	1.13	1.37	<0.0001
	Semaglutide vs. Vehicle	0.89	0.83	0.95	0.0002
Mac-2 area	Vehicle vs. Control	3.8	1.0	14.7	0.0539
	Semaglutide vs. Vehicle	0.33	0.13	0.83	0.0135
Osteopontin area	Vehicle vs. Control	11.3	2.6	48.6	0.0004
	Semaglutide vs. Vehicle	0.43	0.16	1.16	0.1110

The area of tunica media was significantly increased in vehicle-treated mice compared to control in study A (*p* < 0.0001) and study B (*p* = 0.0137) (Fig. 3D and E). In semaglutide treated mice, the media area was increased compared to the control-treated mice in Study A (*p* = 0.0097). Furthermore, the media area was lower in the semaglutide treated group than in the vehicle treated group, albeit this difference did not reach significance (Fig. 3D). In Study B, the media area was decreased in semaglutide treated mice compared to the vehicle group (*p* = 0.0025) (Fig. 3E). The tunica media area (22384 μm^2^ ± 2018) did not increase in mice with application of a cuff alone for 6 h (Fig. 3F) compared to the tunica media area in the control group in Study A (33609 μm^2^ ± 5671) and Study B (35712 μm^2^ ± 8272) (Fig. 2D and E). In a combined analysis of Study A and Study B (see Table 1), there was a significant reduction of the media area of ≈11% in semaglutide dosed animals compared to vehicle (*p* = 0.0002). Furthermore, there was a significant increase in tunica media area of ≈24% in vehicle-dosed animals compared to the control group (*p* < 0.0001). In Supplementary Figs. 8–10, details of tunica media area versus depth (slide number) are visualized for individual animals in Study A, B and the control study.

### 3.4 Electric injury resulted in a dramatic increase in osteopontin expression in vascular smooth muscle cells

**Fig. 4:**
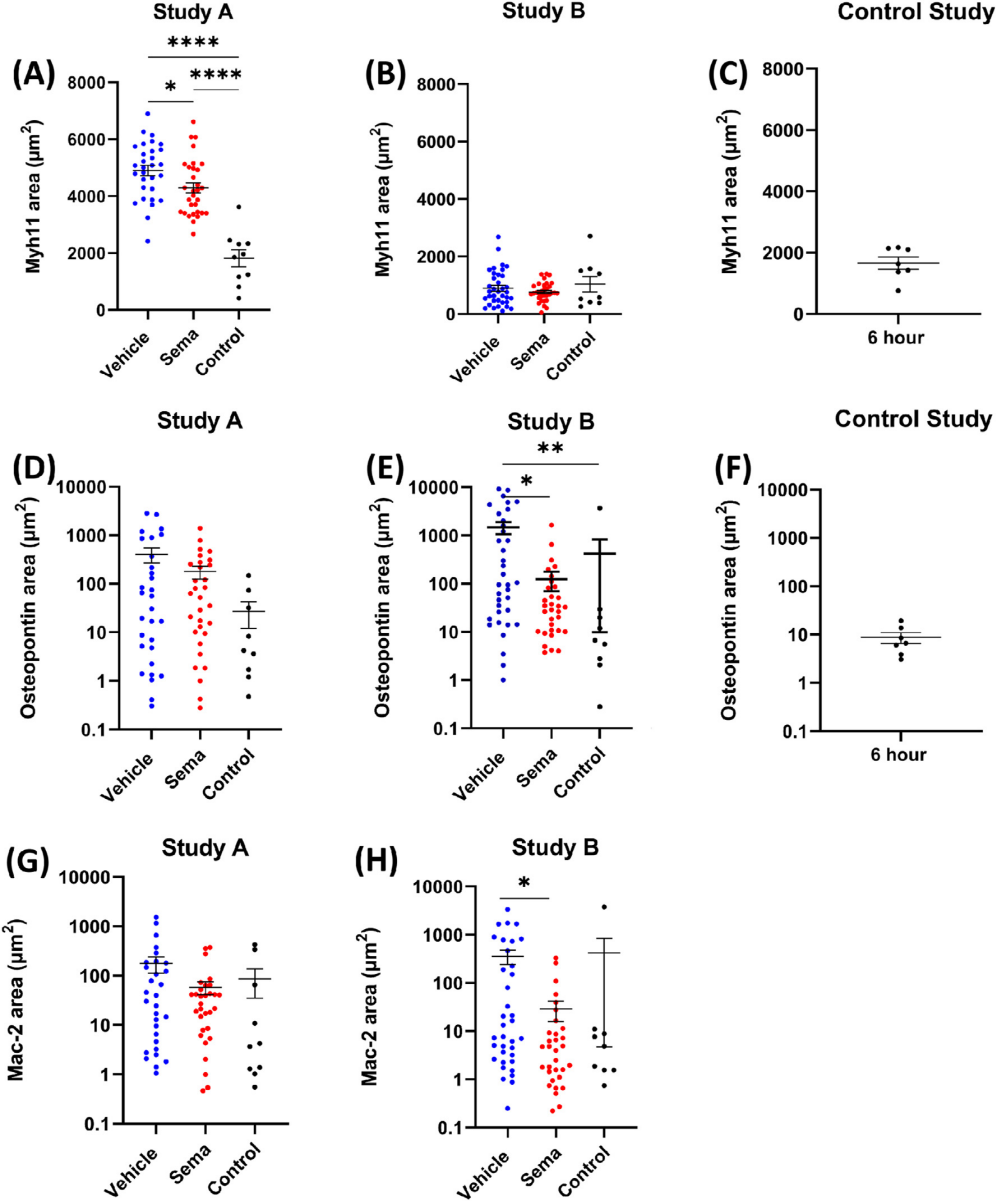
Area of Myh11, osteopontin and Mac-2. (A) Myh11 area in mice from Study A (electric injury). (B) Myh11 area in mice from Study B (plaque erosion study). (C) Myh11 area in mice from the control study (constrictive cuff). (D) Osteopontin area in mice from Study A (electric injury). (E) Osteopontin area in mice from Study B (plaque erosion). (F) Osteopontin area in mice from the control study (constrictive cuff). (G) Mac-2 area in mice from Study A (electric injury). (H) Mac-2 area in mice from Study B (plaque erosion study). Myh11 and osteopontin area was quantified by ISH duplex staining of approximately 10 slides in total per animal (every 50th stained slide). Mac-2 area was quantified by IHC staining of approximately 10 slides in total per animal (every 50th stained slide). Each data point represents one animal. Statistical significance:∗*p* < 0.05, ∗∗*p* < 0.01, ∗∗∗∗*p* < 0.0001. Results are shown as mean ± SEM. Note that the y-axis is log scale in (D), (E), (F), (G) and (H).

Fig. 4 shows the area of Myh11 and osteopontin in the tunica media and tunica intima of each LCCA. Four weeks after electric injury of the LCCA, Myh11 expression was significantly higher in vehicle (*p* < *0.0001)* and semaglutide (*p* < 0.0001) dosed animals compared with controls in study A (Fig. 4A). Furthermore, the expression of Myh11 was reduced in the group treated with semaglutide compared with the vehicle treated group (*p* = 0.0459). Four weeks after electric injury of the LCCA and application of a cuff for 6 h before termination (Study B), Myh11 expression was not statistical significantly changed in vehicle and semaglutide-dosed animals compared to the control group (Fig. 4B). The Myh11 area in LCCA sections from animals where a cuff had been applied alone for 6 h was fully comparable to the control groups in study A and B (Fig. 4C). No significant differences in osteopontin levels were found between the groups in the electric injury study, however, nonsignificant trends towards increased areas in vehicle- and semaglutide treated animals were observed (Fig. 4D). In Study B, the expression of osteopontin was increased in vehicle-dosed animals compared with control (*p* = 0.0036) and semaglutide dosed animals (*p* = 0.0384) (Fig. 4E). A combined analysis of Study A and B on osteopontin levels showed a significant increase (*p* = 0.0004) in the vehicle group compared with the control group of ≈11.3 fold, see Table 1. When comparing the semaglutide treated group with the control group, a non-significant trend towards ≈57% reduction in the semaglutide treated group was observed (Table 1). In the control study, 6 h with cuff alone resulted in osteopontin areas comparable to the control groups in study A and B, i.e., application of a cuff for 6 h did not influence osteopontin levels (Fig. 4F). Plasma osteopontin levels and coagulative markers D-dimer and platelet factor 4 (PF4) was measured in animals from Study B. There were no differences between groups (Supplementary Fig. 11).

### 3.5 Semaglutide reduced infiltration with Mac-2 positive cells in the tunica intima and media

Fig. 4 shows the area of Mac-2 in study A and B (plaque erosion). In study A, there were no significant differences in Mac-2 area between the groups (Fig. 4G). In Study B, the Mac-2 area was significantly decreased in semaglutide treated animals compared with the vehicle group (*p* = 0.0107, Fig. 4H). In a combined analysis of study A and Study B, there was a borderline significant increase of Mac-2 area of ≈3.8-fold in the vehicle-treated group compared with control (*p* = 0.0539), and treatment with semaglutide reduced the Mac-2 area significantly with ≈67% (*p* = 0.0135, Table 1).

### 3.6 GLP-1 receptor expression in LCCA

**Fig. 5:**
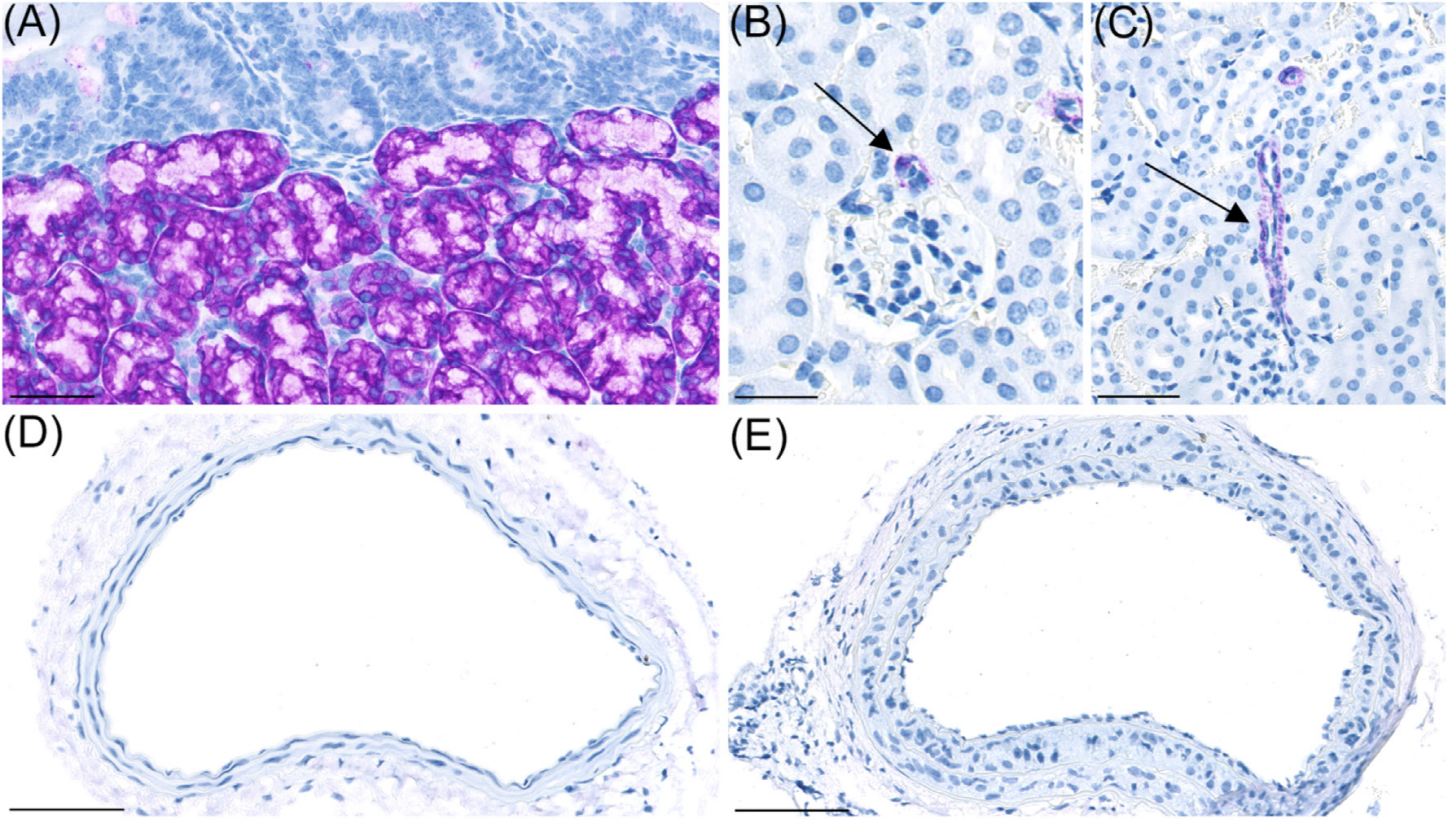
GLP1-R staining. (A) GLP1-R (purple) was detected at high levels in Brunner's gland in duodenum. Scalebar = 50 μm. (B) GLP-1R was also expressed in arterioles supplying glomeruli in mouse kidney (arrow). Scalebar = 50 μm. (C) GLP1-R was also expressed in larger arterioles of mouse kidney (arrow). Scalebar = 50 μm. GLP-1R expression was not observed in carotid artery without neointima (D) or in carotid artery with neointima formation (E). Scalebar in panel (D) and (E) = 100 μm.

Representative images of GLP-1R IHC applied on histological sections of duodenal Brunner's glands, kidney and LCCA with or without neointima are shown in Fig. 5A–E. In good agreement with previous reports, Brunner's gland displayed robust staining for GLP-1R (Fig. 5A) and GLP1R was also detected, at lower levels, in kidney arterioles (Fig. 5B–C) [20,21]. GLP-1R expression was not observed in LCCA sections with or without neointima (Fig. 5D–E).

### 3.7 GLP-1 receptor expression in human coronary vessels with and without atherosclerotic plaque development

Spatial transcriptomics, *in situ* hybridisation, and immunohistochemistry performed on human coronary vessels with and without atherosclerotic plaque development showed complete absence of GLP-1R expression (Supplementary Figs. 12 and 13).

### 3.8 Semaglutide does not attenuate human coronary artery smooth muscle cell proliferation *in vitro*

Semaglutide treatment did not affect proliferation of hCASMCs exposed to starvation, PDGF-BB, and full medium (Supplementary Fig. 14).

## 4 Discussion

Here, we show that semaglutide treatment reduced vessel remodelling in a murine model recapitulating mechanisms related to VSMC function described in human atherosclerosis. This effect appears to be driven by anti-inflammatory and -proliferative mechanisms independent of receptor activation/signalling in the resident vascular cells.

Perivascular application of an electrical current has previously been described to cause severe VSMC injury and death, denudation of the endothelium and platelet-rich mural thrombosis. Following degradation of the thrombus, inflammatory cells infiltrate the vessel wall to remove the necrotic debris, whereas VSMCs in the border zones proliferate and migrate to re-populate the media and accumulate in intima [22]. In the present study, semaglutide treatment very potently reduced the intimal (∼66%) and medial (∼11%) areas as evidenced from the combined analysis of study A and B. Within this context, the transient loss of contractile protein expression observed after vascular injury is followed by re-establishment of the contractile phenotype after vessel repair [22,23]. Hence, the present finding of lower expression of the contractile marker Myh11 in animals treated with semaglutide agrees with an attenuation of VSMC proliferation upon injury to the vessel.

The pharmacological effect on vascular remodelling observed in the present study is in line with previous experiments that found an attenuating effect of the GLP-RAs exendin-4 [24,25] and liraglutide [26] on intimal hyperplasia induced by intravascular wire/balloon. Notably, in these studies the animals were either wildtype [24,25] or diabetic [26] whereas the ApoE−/− mice used in the present study have normal glucose levels but increased levels of lipids and develop atherosclerotic lesions at susceptible vascular sites even on a chow diet. This atheroprone phenotype of the mice share key similarities with human disease as it has been observed in both human and murine atherosclerotic plaques that contractile VSMCs recruited from the tunica media undergo phenotypic conversion to proliferative synthetic cells [2,18]. These cells then migrate into the developing atherosclerotic plaque where they may adopt alternative phenotypes that could contribute both positively and negatively to disease progression [2]. Whether the anti-proliferative effects of semaglutide is a mechanism involved in the cardiovascular risk reduction in patients remain to be established, but it is tempting to speculate that attenuation of VSMC proliferation, in a local environment that favours plaque de-stabilising phenotypes, is beneficial. Clearly, more pre-clinical and clinical evidence is needed to support such a mechanism of action.

There is accumulating pre-clinical (e.g. Ref. [27]) and clinical [28,29] evidence supporting that the reductions in adverse cardiovascular events with GLP-1RAs may—at least in part—be driven by anti-inflammatory mechanisms. In line with this mode of action, a reduction in the pro-inflammatory cytokine osteopontin and macrophage marker Mac-2 was detected in the vessel wall in the present study. Interestingly, this effect of semaglutide treatment appeared more pronounced in study B than in study A. Here, the non-significant effects of semaglutide in study A may reflect that inflammation in the vessel wall was resolved at week 4 [22] and that placement of the cuff was needed to induce an acute inflammatory response at this timepoint. Although we observed that application of a cuff alone for 6 h to a vessel without electric injury did not influence osteopontin or Mac-2 levels, it has previously been described that induction of downstream oscillatory shear stress with a constrictive cuff resulted in an acute pro-inflammatory vascular response as evidenced by infiltration of neutrophils (Ly6G+ cells) into the intimal area [17]. It is therefore possible that the acute inflammatory changes in the vessel wall were more exacerbated in study B than study A, and that this allowed for detection of a more pronounced effect of semaglutide.

The anti-inflammatory effect of semaglutide does not appear to be mediated by a direct effect on vascular cells as GLP-1Rs were not expressed in any of the wall resident cells. This lack of receptor expression is supported by published observations in atherosclerotic lesions of mouse [27] and human [3] arteries as well as the herein reported lack of expression in human coronary vessels with and without atherosclerotic plaques. Furthermore, treatment with semaglutide did not affect proliferation of primary VSMCs (see supplementary data). An indirect effect may relate to the lowering of plasma cholesterol induced by semaglutide treatment as exposure to aggregated and oxidised LDL is known to induce a switch to macrophage-like VSMCs or foam cells characterised by CD68, LGALS3, and pro-inflammatory cytokine expression [30]. However, it should be noted that anti-atherosclerotic and -inflammatory effects of GLP-1RA treatment are evident in the absence of cholesterol-lowering in murine models [27] as well as humans [7,8], suggesting that other non-LDL mechanisms such as triglyceride lowering and anti-inflammatory effects are also at play. Lowering of inflammation has been documented in numerous large randomized clinical trials with long-acting GLP-1RAs [31]. A clinical trial is ongoing which investigates the effect on inflammation in carotid arteries (https://clinicaltrials.gov/ct2/show/NCT04032197). This trial also investigates fibrous cap thickness and lipid rich necrotic core volume, which may help to further clarify how semaglutide treatment affects plaque morphology and mechanisms precipitating cardiovascular events.

In the present study, we utilised a protocol previously reported to recapitulate certain features associated with superficial erosion, including arterial injury and loss of endothelial cells in regions of disturbed flow [17,32]. However, despite obtaining similar increases in the neointimal area following electrical injury, placement of the constrictive cuff did not induce denudation of the endothelial layer located at the healed site. The preservation of an intact endothelium and absence of thrombi generation was further supported by the unaltered plasma levels of PF4 and d-dimer. What underlies this discrepancy is unclear as great attention was given to fully replicate the protocol including using the same animal vendor, bipolar microcoagulator, and cone-shaped polyethylene cuffs.

Following percutaneous coronary intervention there is a risk of restenosis and late stent thrombosis. Although the present model does not directly resemble the injury in patients undergoing vascular reconstructions, VSMC phenotypic modulation and induction of proliferation are also observed in response to vascular injury and underlies cell accumulation leading to restenosis after stenting [33]. Hence, future studies should focus on the extent to which semaglutide treatment may affect the outcome for patients undergoing vascular interventions.

In conclusion, the present study demonstrates that semaglutide treatment reduces intimal and medial hyperplasia following electrical injury and blood flow perturbation in an atheroprone mouse model, an effect that may be driven by anti-inflammatory and -proliferative mechanisms that are independent of GLP1-R activation or signalling in vascular cells. These pharmacological effects of semaglutide treatment may provide a mechanistic basis for the cardiovascular risk reduction.

## Financial support

This study was supported by a grant from the LifePharm Centre of In Vivo Pharmacology.

## Author contributions

The study was designed by DMJ, JFB, MFBB, BR, GFS, JL, and MN. Histological supervision was done by LMV, MKEO, CP, RKK, and HH. DMJ, MFBB, JCB, and AU performed the experimental work. Initial data was analysed by DMJ, following data interpretation by all authors. DMJ and MN drafted the manuscript, which was edited and critically revised by all authors. LBK contributed to the manuscript writing. The final manuscript was read and approved by all authors.

## Data availability

Additional data are available on a reasonable request from the corresponding author.

## Declaration of competing interest

The authors declare the following financial interests/personal relationships which may be considered as potential competing interests: The authors BR, LMV, MKEO, JCB, AU, CP, RKK, HH, LBK and MN are employees at Novo Nordisk. Novo Nordisk markets semaglutide for the treatment of diabetes and obesity. DMJ, GFS, and JL are present or former employees at University of Copenhagen and have collaborated with Novo Nordisk on this project.
