# Supplementary material for: Semaglutide treatment attenuates vessel remodelling in ApoE−/− mice following vascular injury and blood flow perturbation
Source: Atheroscler Plus. 2022 Jun 4;49:32–41. doi: 10.1016/j.athplu.2022.05.004 (PMC9833261; doi:10.1016/j.athplu.2022.05.004)
Supplement: Multimedia component 1 [file mmc1.docx]

**Submission declaration**

- I confirm that the article is not under consideration for publication elsewhere.
- I confirm that the article is approved by all authors and tacitly or explicitly by the responsible authorities where the work was carried out.
- I confirm that if the article is accepted, it will not be published elsewhere by the authors, including electronically in the same form, in English or in any other language, without the written consent of the copyright-holder.

On behalf of all the authors,


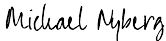


Michael Nyberg. MSc, PhD
