# Supplementary material for: Semaglutide treatment attenuates vessel remodelling in ApoE−/− mice following vascular injury and blood flow perturbation
Source: Atheroscler Plus. 2022 Jun 4;49:32–41. doi: 10.1016/j.athplu.2022.05.004 (PMC9833261; doi:10.1016/j.athplu.2022.05.004)
Supplement: Multimedia component 2 [file mmc2.docx]

**Supplementary Material**

**Semaglutide treatment attenuates vessel remodelling in ApoE-/- mice following vascular injury and blood flow perturbation**

Ditte Marie Jensen^a,b,^ , Gry Freja Skovsted^a^, Mathilde Frederikke Bjørn Bonde^b^, Jacob Fog Bentzon^c,d^, Bidda Rolin^b^, , Grégrory Franck^e^, Maria Katarina Elm Ougaard^b^, Louise Marie Voetmann^b^, Julian Christoffer Bachmann^b^, Anna Uryga^b^, Charles Pyke^b^, Rikke Kaae Kirk^b^, Henning Hvid^b^, Lotte Bjerre Knudsen^b^, Jens Lykkesfeldt^a^, Michael Nyberg^b^

^a^Department of Veterinary and Animal Sciences, University of Copenhagen, Ridebanevej 9, 1870 Frederiksberg C, Copenhagen, Denmark; ^b^Research and Early Development, Novo Nordisk A/S, Novo Nordisk Park 1, 2760 Maaloev, Denmark; ^c^Department of Clinical Medicine, Heart Diseases and Steno Diabetes Center Aarhus, Palle Juul-Jensens Boulevard 99, Aarhus University, 8200 Aarhus, Denmark, ^d^Centro Nacional de Investigaciones Cardiovasculares (CNIC), Alle Melchor Fernandez Almagro, 3, 28029, Madrid, Spain , ^e^INSERM U1148, Laboratory for Vascular Translational Science, Bichat Hospital, 46 Rue Henri Huchard, Paris, France.

**Supplementary Methods**

*Administration of semaglutide*

Following the acclimatization period, animals in Study A and B were administered once daily subcutaneously (s.c.) injections using a NovoPen®, of either vehicle (50 mM phosphate 70 mM sodium chloride, 0.007% polysorbate 20, pH 7.4, Novo Nordisk, Måløv, Denmark) or semaglutide (3 nmol/ml in vehicle, pH 7.4, Novo Nordisk, Måløv, Denmark). Injections were done at the same time a day. To avoid excessive nausea and weight loss in the semaglutide treated group, doses were titrated over 15 days, starting at 3 nmol/kg at day 1-3, 6 nmol/kg at day 4-8, 9 nmol/kg at day 9-11, 12 nmol/kg at day 12-14 to the final dose of 15 nmol/kg at day 15 until termination. The dosing volume in the vehicle-treated mice was gradually increased accordingly. Mice were weighed daily during the titration period for adjustment of dose volume. Daily dosing of semaglutide or vehicle continued throughout the whole study period.

*Surgical procedures on the LCCA*

All surgical procedures were performed under anaesthesia with isoflurane (2% in N_2_O/O_2_ (70%/30%)), and only after complete absence of reflexes. In short, the left carotid artery was identified and bluntly dissected free from connective and fat tissue. After that, either vascular injury and/or application of a perivascular constrictive cuff as done by Franck et al. [1] was performed, according to the protocol. The surgical procedure was done by placing the animal in the supine position, head proximal to the surgeon, in a mask with continuous supply of isoflourane, on an operation table positioned on top of a heating mat (37°C), under a stereomicroscope. Eye ointment was applied to avoid eye damages and hair machine removed from the neck and throat. A longitudinal skin incision was made overlying the thyroid bone (~1-1.5 cm in length). The sternomastoid and omohyoid muscle to the left of the thyroid bone was retracted to expose the carotid sheath, which was opened, and the LCCA identified. The LCCA was bluntly dissected free until the bifurcation of the external- and internal carotid artery. Either electric injury and/or application of a perivascular constrictive cuff were thereafter performed on the LCCA. After surgery, the skin was closed with surgical staples (BD Autoclip), and mice were given s.c. NaCl (0.5 ml, 37°C) to avoid dehydration. For study A and B, analgesia (buprenorphine 0.2 mg mixed in 1 g Nutella) was administered by voluntary oral ingestion (dose of 1 mg/kg body weight) one hour before surgery and subsequently twice daily for two days after surgical procedures, following the recommendations in Abelson et al. [2]. Animals in Study C were administered s.c. temgesic (buprenorphin 0.3 mg/ml) 0.05 mg/kg (30 min pre-surgery and 5-8 hours post-surgery) and rimadyl (5 mg/kg) (30 min pre-surgery and thereafter 24- and 48-hours post-surgery).

*Vascular injury*

Vascular injury was induced by electric injury of the LCCA, as previously described [1,3,4]. With the forceps tips of a VIO50 burner device (Erbe, Germany), an electric current (5 watts for 8 seconds) was applied to the LCCA, inferior to the carotid bifurcation. The procedure was repeated twice, moving the forceps tips away from the bifurcation for a total distance of approximately 2-4 mm. Mice from the control group were sham operated following the same procedure as above, but without applying an electric impulse on the LCCA.

*Induction of turbulent flow*

Four weeks after electric injury surgery, mice in study B underwent surgery to apply a perivascular constrictive cuff to induce local flow perturbation [1]. The cuff was manufactured in polyethylene by 3-dimensional stereolithography printing (Proto Labs, UK), as used in [3,5]. The cuff was applied to the LCCA distal to the previously electric injured area, see Supplementary Figure 2. The cuff consists of a hollow tube cut in two pieces on the longitudinal axis, resulting in a large (2/3 diameter) and a small (1/3 diameter) piece. The large part of the cuff was applied under the LCCA, and the smaller part of the cuff was applied on top of the artery. The cuff was then ligated with a silk suture. The internal diameter of the cuff decreases gradually to become constrictive at its end (distal diameter: 250 µm and proximal diameter: 500 µm). Mice from the control group were sham operated following the same procedure as above, but without applying the constrictive cuff and an electric impulse on the LCCA.

*Termination*

Mice were anesthetized with isoflurane (4% in N_2_O (70%)/ O_2_ (30%)). The thoracic cavity was cut open, and the heart exposed. The left ventricle was perfused (5 ml/min for 2 min) with ice-cold NaCl followed by 10% neutral buffered formalin (NBF). The LCCA from the aortic arch and the bifurcation (external- and internal carotid artery) was isolated and dissected free of fat and connective tissue, put on tissue paper (Micropore), and stored in 10% NBF until processing, paraffin embedding and histological examination.

*Blood collection and measurements*

Blood samples at baseline and termination were collected for Study A and B. Baseline blood samples were collected two days before study start by punctuation of the vena sublingualis in (100 µl) EDTA coated vials (Microvette, Sarstedt, Germany). Terminal blood samples were collected after animals were anesthetised by punctuation of the retro-orbital sinus using a hematocrit tube (Na-Heparin-coated). For collection of plasma, blood was transferred to EDTA-K2-coated vials (MILI041-TOM-14C, Milian, Microvette, Sarstedt, Germany), and plasma was separated (4°C, 8000 RPM, 5 min within 20 min) and stored at -20°C. Total cholesterol and triglycerides were measured on a COBAS 6000 multi-analyser according to the manufacturer’s recommendation (Roche Diagnostics).

*Coagulation analysis*

Plasma from terminal blood samples in Study B were used to determine coagulative markers, D-dimer and platelet factor 4 (PF4), and osteopontin. D-dimer in plasma was measured using a kit (Abbexa, catalogue no. abx258705), based on the competitive ELISA method, following the manufacturer’s instructions. PF4 was determined using a PF4 quantikine sandwich ELISA kit (RnD Systems, catalogue no. MCX400), following the manufacturer’s instructions. Osteopontin in plasma was measured using a sandwich ELISA assay (R&D systems, catalogue no. MOST00).

*Histological examination*

After fixation in 10% NBF, the LCCA was trimmed, processed and infiltrated with paraffin overnight. Subsequently, the tissue was embedded in blocks of paraffin containing 15-20 vessels per block. The position of each vessel in the block was randomised, and a piece of liver, kidney or lung tissue was used for orientation. The cut surface was closest to the bifurcation end. Sections of 4.5 µm thickness were cut on a microtome and mounted on Superfrost Plus slides (ThermoFisher Scientific), starting with slide 1. A total of approximately 2 mm of the LCCA was sectioned, yielding approximately 500 slides per LCCA, see Supplementary Figure 2 for details. Slides were de-paraffinated in xylene and rehydrated in a series of graded ethanol to tap water and subjected to different staining methods. Details of the key reagents used for the various histological staining are described in Supplementary Table 1.

To identify the tunica intima and tunica media, every 10^th^ section (starting at section 1) was stained with Movat’s Pentachrome (Abcam, Cambridge, UK) according to the manufacturer’s instructions.

For identification of macrophages, every 50^th^ LLCA sections was stained with a monoclonal rat anti-mouse Mac-2 antibody (Biolegend, San Diego, CA, USA) using the automated Ventana Ultra Discovery system (Roche Diagnostics, Hvidovre, Denmark) and the appertaining reagents (all from Roche Diagnostics). In brief, the slides were baked, deparaffinised and pre-treated for antigen retrieval in CC1 buffer (Tris-EDTA buffer pH 7.8). Then endogenous peroxidase activity was blocked with Discovery inhibitor, and unspecific antibody binding was blocked with TNB blocking buffer prior to incubation with the primary antibody diluted 1:2000 for 60 min. The primary antibody was subsequently detected with a HRP-labelled anti-rat multimer (OmniMap anti-rat HRP, Roche) and visualised using DAB chromogen. Slides were counterstained with hematoxylin (Sigma-Aldrich, Brøndby, Denmark), and cover glasses were mounted with Pertex (Sigma-Aldrich).

Detection of CD31 by IHC was done as described above for Mac-2, except that a monoclonal rabbit anti-mouse CD31 antibody (Cell Signaling Technology, Danvers, MA, USA) was used as the primary antibody and the HRP-labelled BrightVision anti-rabbit polymer (Roche) was used for detection of the primary antibody.

For detection of VSMCs and exploration of possible neointima formation and vascular remodelling, the mRNA expression levels of myosin heavy chain 11 (Myh11) and osteopontin (SSP1), respectively, were assessed with in situ hybridization (ISH) on every 50^th^ section, using RNAscope probes (Advanced Cell Diagnostics (ACD), Newark, CA, USA, see table X). The ISH was performed as a RNAscope duplex assay with simultaneous detection of two different mRNA signals. The experimental procedures were performed fully automated on a Ventana Ultra Discovery system (Roche Diagnostics) according to the manufacturer’s instructions. In brief, slides were baked, deparaffinized, pre-treated (i.e., heat-induced target retrieval followed by inhibitor and protease treatment, all reagents from ACD), and ISH was then performed using the RNAscope VS Universal AP kit with the red and teal detection kit (ACD). ISH with a positive control RNAscope probe for mouse PPIB and a negative control RNAscope probe for bacterial DapB were included in each run. Slides were finally counterstained with hematoxylin and coverglasses mounted with Pertex (Sigma-Aldrich).

Stained sections were scanned on an Olympus VS120 scanner (Olympus, Hamburg, Germany) or Hamamatsu Nanozoomer S60 scanner (Hamamatsu, Hamamatsu City, Japan) for the generation of digital whole slide images (WSI). Image analysis was then performed to quantify areas of interest and areas with positive staining using the software VIS (Visiopharm, Hørsholm, Denmark). In the WSI of Movat’s pentachrome-stained sections, a pre-trained image analysis algorithm was used to identify and quantify the areas of a) the tunica interna (delineated by the vessel lumen and the inner elastic lamina) and b) the tunica media (delineated by the inner elastic lamina and the outer elastic lamina), as shown on Supplementary Figure 3. In the WSI of sections stained by IHC or ISH, the areas with positive staining (identified by the chromogens DAB, purple, red, or teal) were quantified in the region in each section delineated by the outer elastic membrane and the vessel lumen, as shown on Supplementary Figure 4, using a threshold-based image analysis algorithm in VIS. The endothelial integrity in WSI of CD31-stained LLCS sections was evaluated by visual inspection.

*Spatial gene expression of human coronary arteries*

Formalin-fixed paraffin-embedded (FFPE) human coronary arteries were obtained from 4 unknown donors, two with and two without atherosclerotic plaque, purchased from AnaBios (California, USA). The quality of RNA extracted with Qiagen RNAeasy FFPE kit (Qiagen, #73504) from one section (10 µm) of each tissue was assessed by a DV200 analysis using Agilent 2100 Bioanalyzer (Agilent Technologies). 1-2 sections of each included sample were collected onto a gene expression visium barcoded slide and stored at room temperature in a sealed box with silica gel for one day. Spatial transcriptomics was performed the day after using the technology from 10x Genomics, called Visium, according to manufactures protocol for hematoxylin and eosin (H&E) staining, imaging, and decrosslinking (10x Genomics, #CG000409, Rev.B) and the protocol for Visium spatial gene expression for FFPE (10x Genomics, CG000407, Rev.C). The H&E-stained tissue sections were scanned using an Olympus VS200 scanner at 20x magnification. Libraries were sequenced using NextSeq550 according to recommendations from 10X Genomics. The sequencing data were processed using Space Ranger (version 1.3), and the data were analysed using the software tool; Loupe browser (ver. 6.0) provided by 10x Genomics.

*In situ* *hybridization and immunohistochemistry of the GLP-1 receptor in human coronary arteries*

Tissue sections of 4.5 µm from the same FFPE coronary arteries employed for spatial transcriptomics mounted on superfrost plus slides (ThermoFisher Scientific, #J1800AMNZ) were used. Fully automated immunohistochemistry and in situ hybridisation of the GLP-1 receptor were performed on the Ventana Ultra Discovery system (Roche Diagnostics International, Switzerland). Slides for in situ hybridisation were baked, deparaffinised, pre-treated (target retrieval, inhibitor, protease, #323250, Advanced Cell diagnostics), hybridised with the Hs-GLP-1 receptor (#519829, Advanced Cell Diagnostics), and enhanced using the RNAscope™ VS Universal AP kit with the VS 2,5 RED reagent kit (#760-236,#760-248, #760-234, Roche). Parallel hybridization of Hs-PPIB (#313909, Advanced Cell diagnostics) and DapB (#312039, Advanced Cell diagnostics) was included serving as positive and negative controls, respectively. Similarly, slides for immunohistochemistry were baked, deparaffinised, pre-treated (CC1, HRP Inhibitor CM, #06414575001, Roche), incubated with Ms-GLP-1R antibody (1:300, In house antibody, Novo Nordisk A/S) for 60 minutes. The signal was enhanced using the ms-HQ system and HQ-HRP kits (#760-4814 and #760-4820, Roche). The antibody was labelled using DAB chromogen (Roche, Cat#760-159). An identical protocol on monkey kidney (IHC) and human pancreas (ISH) was included as positive control tissue, and a protocol without a primary antibody served as a negative control. All slides were counterstained with haematoxylin and bluing reagent (#05277965001; #05266769001, Roche), and coverslips were mounted using Pertex mounting medium. Slides were visualised using Nanozoomer s60 whole slide imaging at 40x magnification (Hamamatsu, Japan).

*Human coronary artery smooth muscle cell cultures*

Human coronary artery smooth muscle cells (hCASMCs) were obtained from Provitro AG. To provide optimal CASMC growth, cells were cultured in smooth muscle cell growth medium (Provitro AG) in a humidified incubator at 37 °C, 5% CO2. Cells were pre-starved for 24h in basal medium (ProVitro AG) + 0.5% FBS (Thermo Fisher Scientific) without supplements (starvation medium) prior stimulation. Cells were then stimulated with 1µM Semaglutide and 20ng/mL PDGF-BB (R&D Systems) for 72h.

*Click-iT EdU cell proliferation assay*

Cells were seeded onto 96 well plate and incubated for 24 h with 10 µM EdU, before being processed with Click-it EdU proliferation assay kit (Thermo Fisher Scientific), according to the manufacturer’s recommendations. Images were captured on the Operetta^TM^ high content analysis system and further processed using Harmony Software 4.1 (Perkin Elmer).

**Supplementary Figure 1. Timeline and overview of Study A, B and the control study**

**
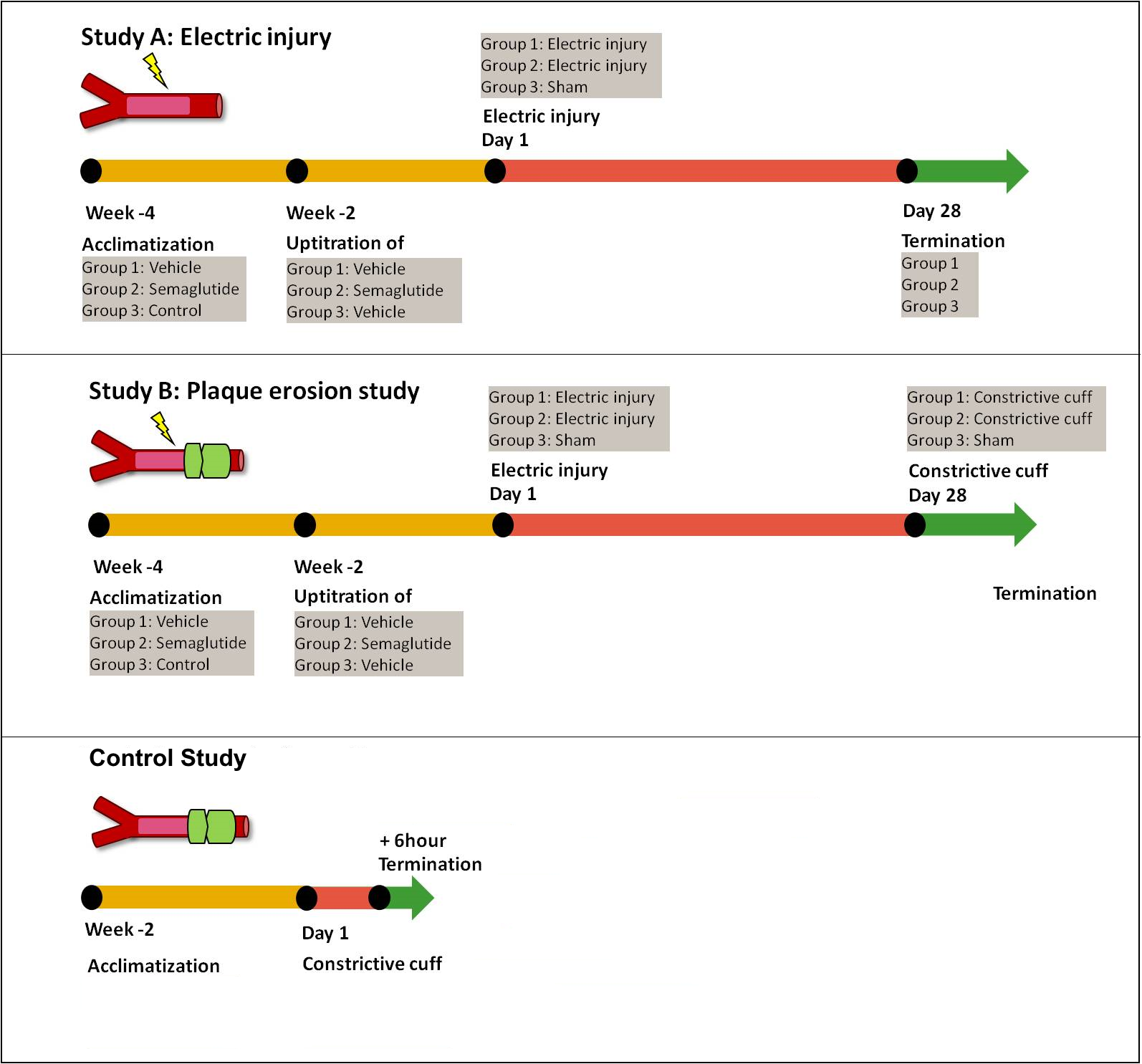
**

**Supplementary Figure 2. Histological sampling of LCCA**

**
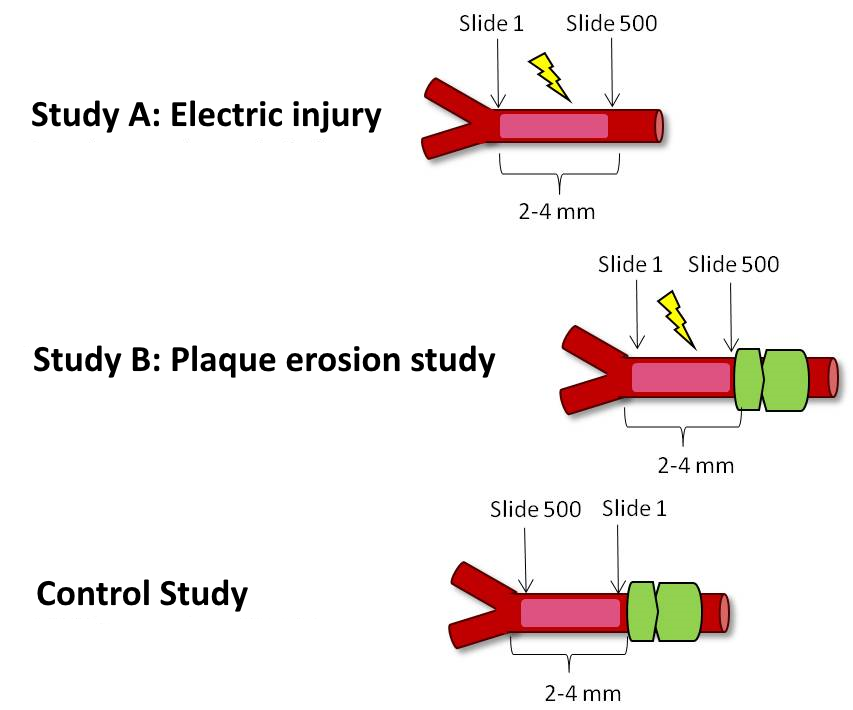
**

**Supplementary Figure 3. Quantification of tunica intima and tunica media area**


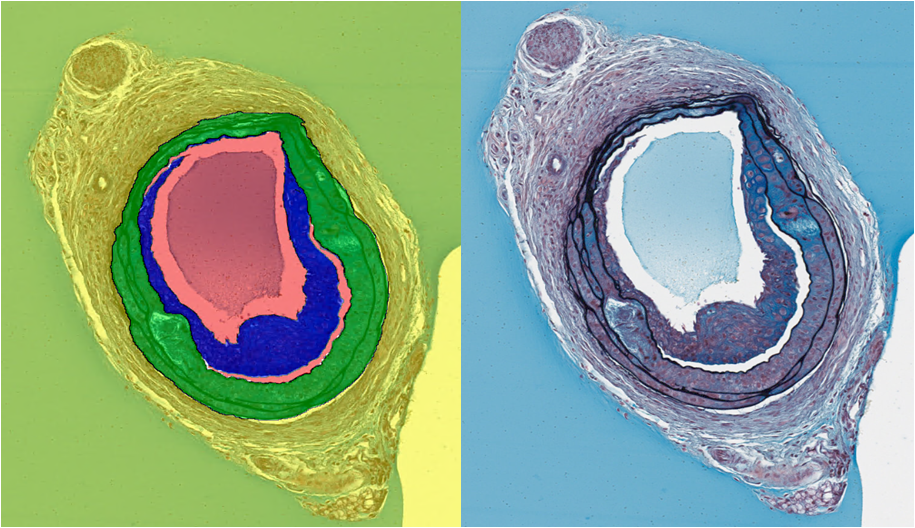


Movat’s pentachrome stained slides (left) was used to train the artificial intelligence (AI) function in the VIS image analysis software (Visiopharm, Hoersholm, Denmark). Yellow colour is background, green colour is tunica media area, blue colour is tunica intima area and red colour is lumen.

**Supplementary Figure 4. Quantification of IHC and ISH positive staining**


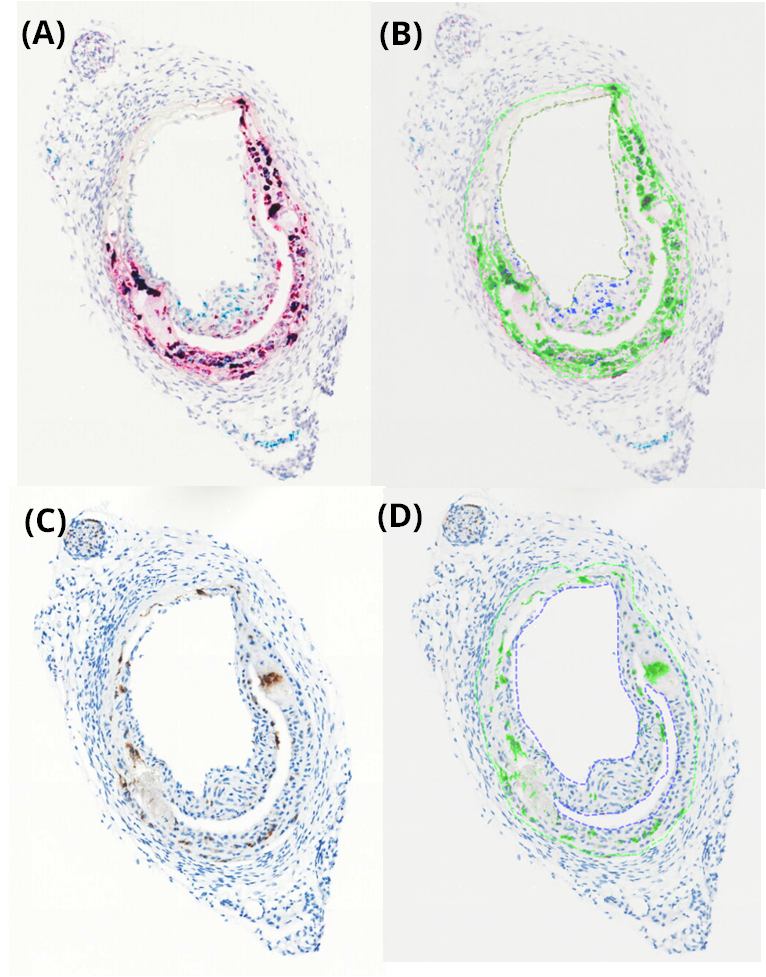


(A) Vessel stained with Myh11 (red) and Osteopontin (teal) by the ISH duplex method. (B) Quantification of the stained areas in tunica media and tunica intima by using the VIS image analysis software. (C) Mac2 staining using IHC and DAB chromogen for visualization. (D) Quantification of the stained areas in tunica media and tunica intima by using the VIS image analysis software.

**Supplementary Figure 5. Tunica intima area versus depth (slide number) for Study A (electric injury)**


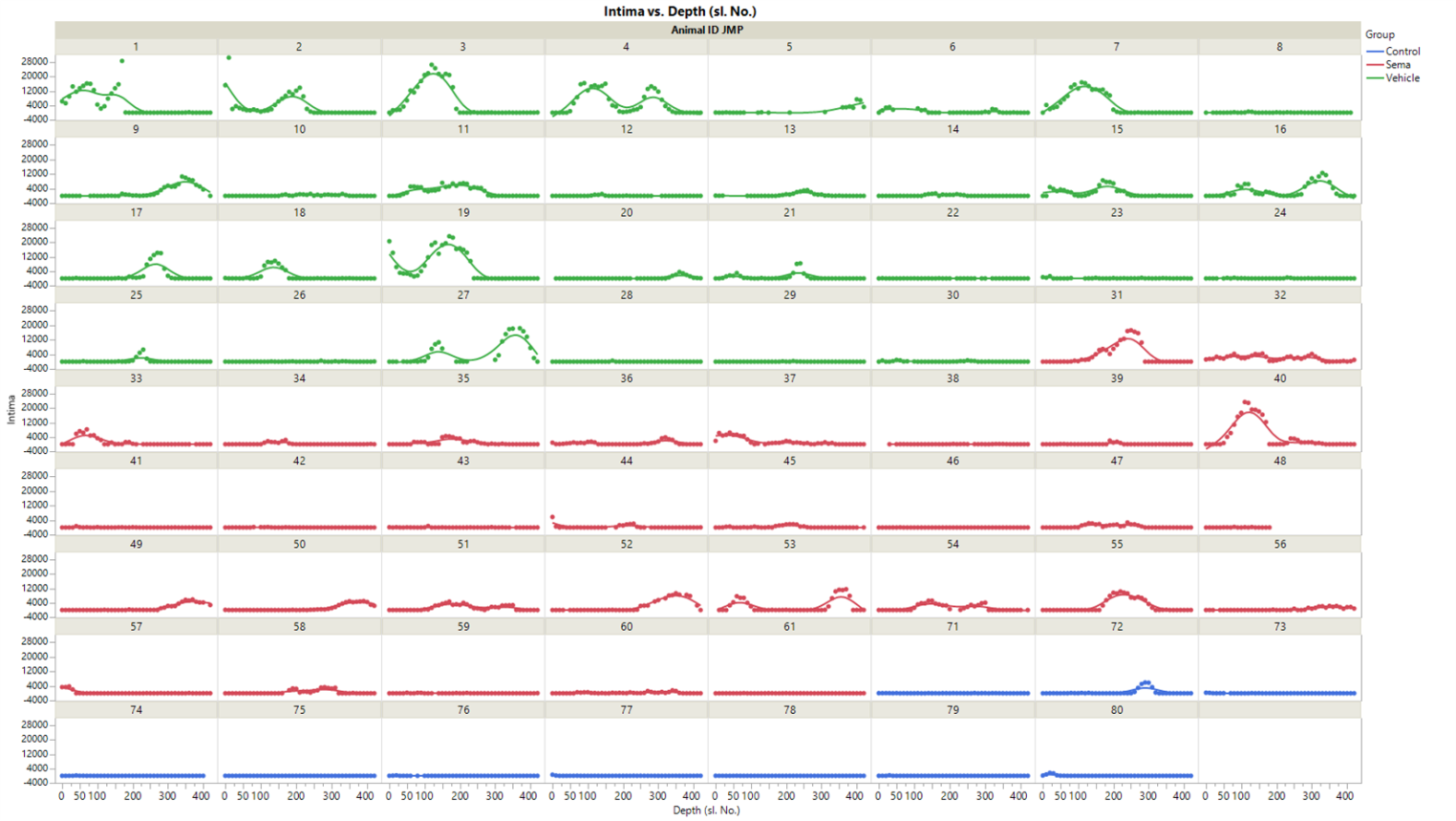


Tunica intima area versus depth of the LCCA (slide number) in individual animals in the vehicle (green), semaglutide (red) and control (blue) groups.

**Supplementary Figure 6. Tuncia intima area versus depth (slide number) for Study B (plaque erosion)**


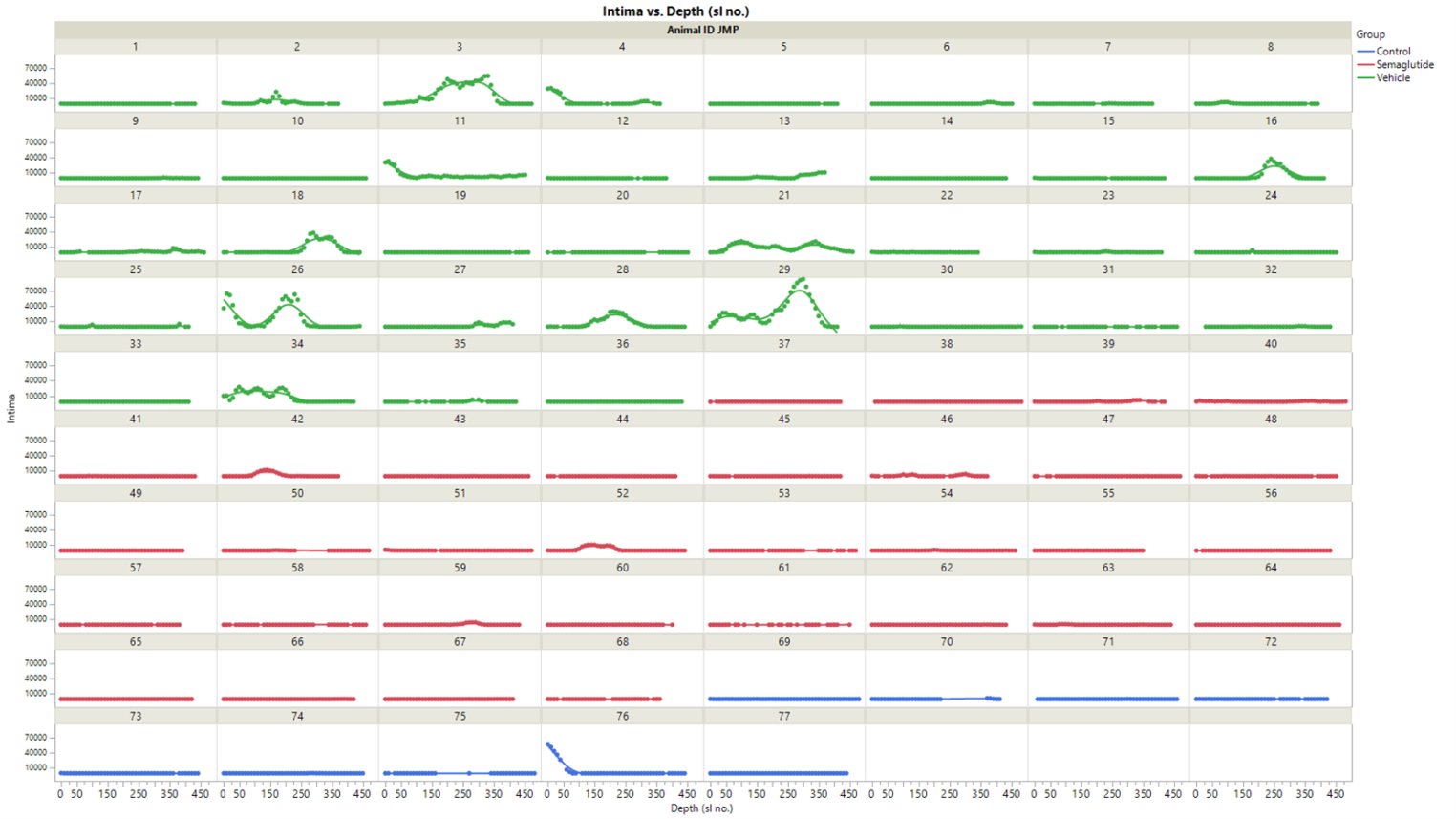


Tunica intima area versus depth of the LCCA (slide number) in individual animals in the vehicle (green), semaglutide (red) and control (blue) groups.

**Supplementary Figure 7. Tunica intima area versus depth (slide number) for the control study (constrictive cuff)**


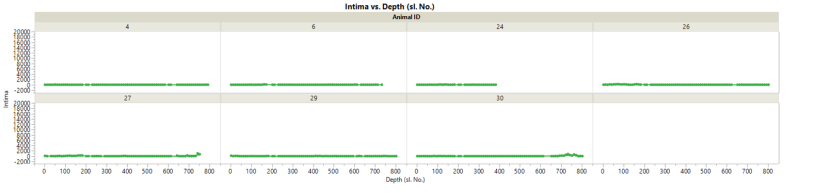


Tunica intima area versus depth of the LCCA (slide number) in individual animals.

**Supplementary Figure 8. Tuncia media area versus depth (slide number) for Study A (electric injury)**


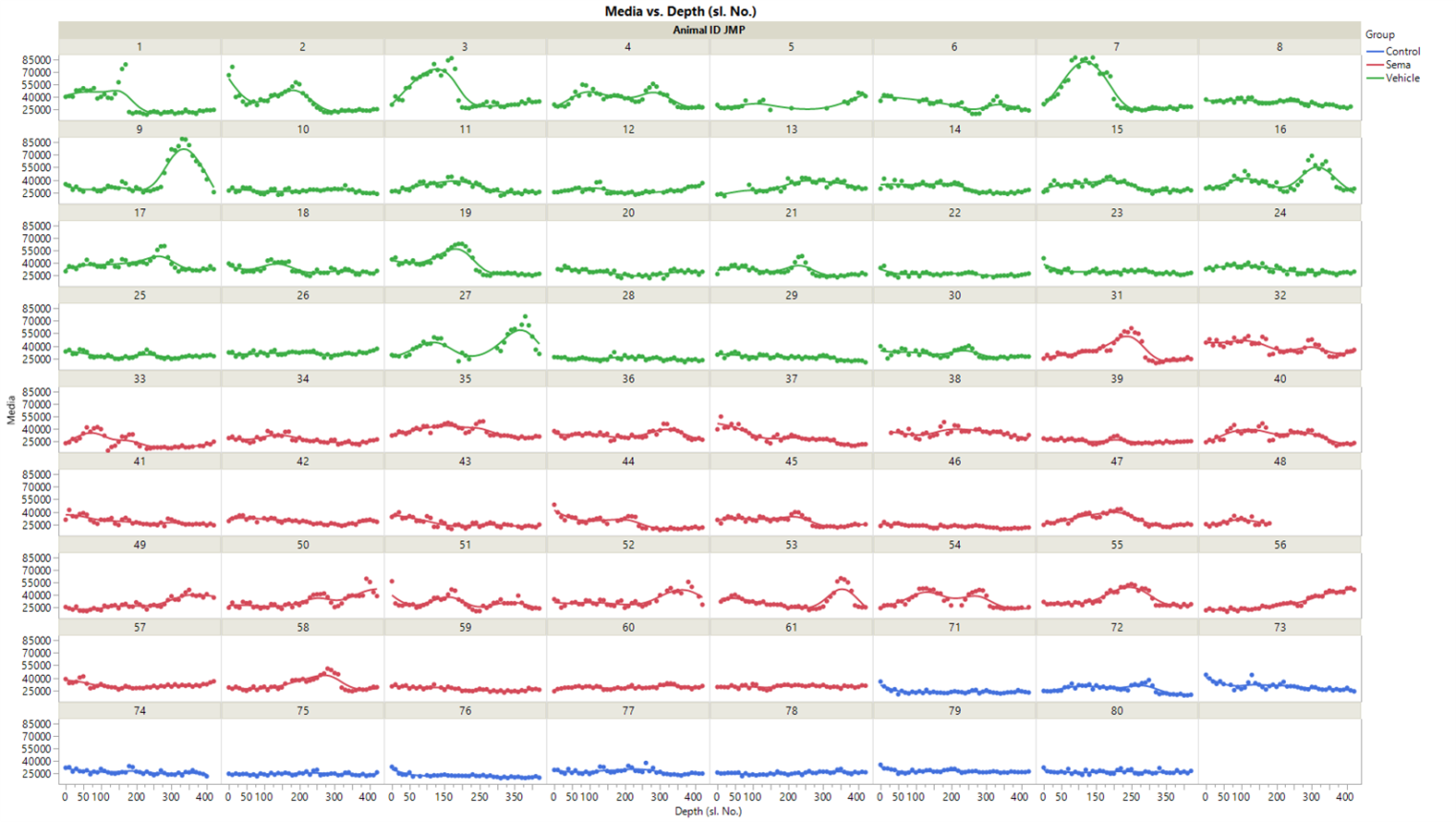


Tunica media area versus depth of the LCCA (slide number), per individual animals in the vehicle (green), semaglutide (red) and control (blue) groups.

**Supplementary Figure 9. Tuncia media area versus depth (slide number) for Study B (plaque erosion)**


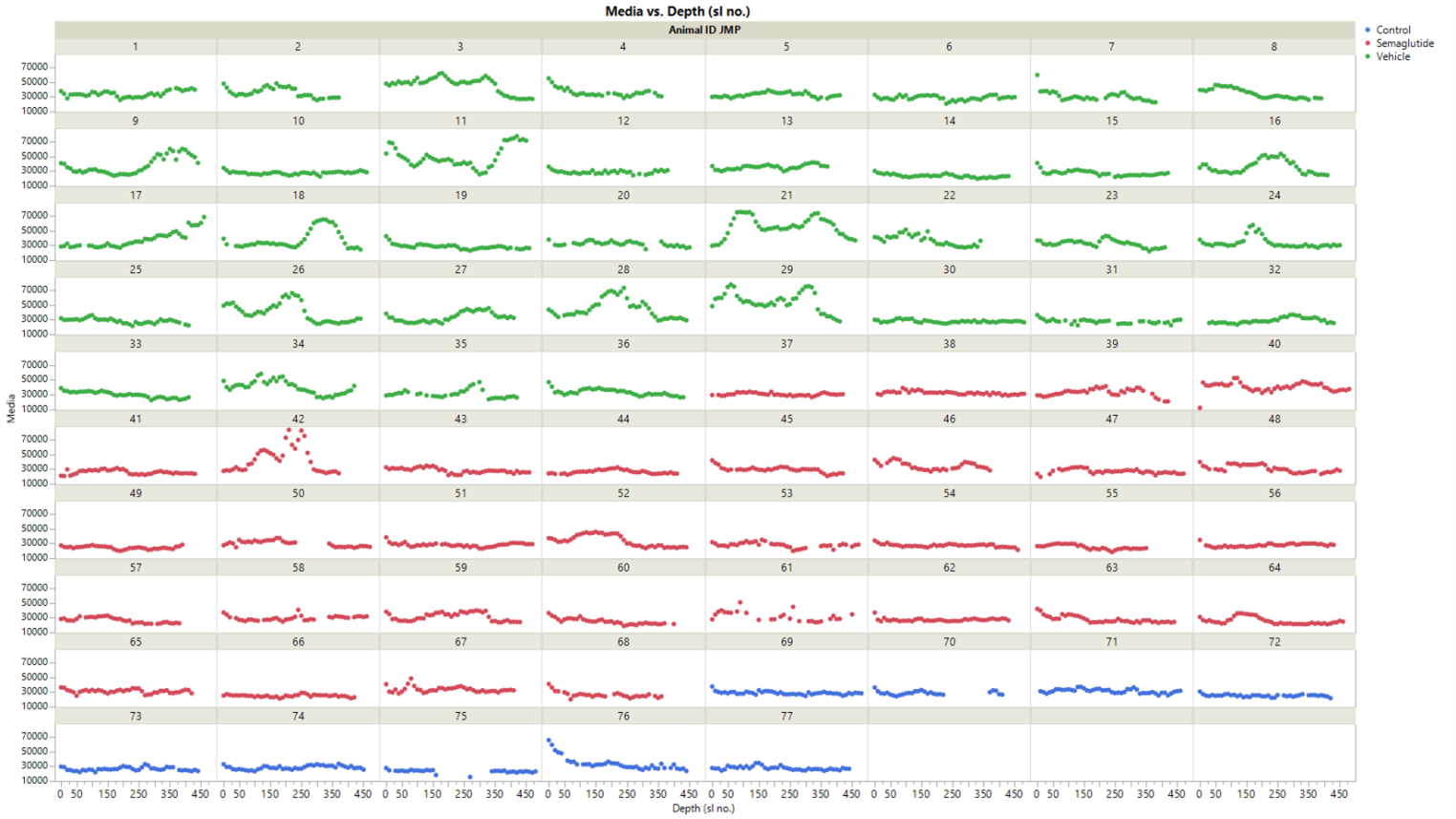


Tunica media area versus depth of the LCCA (slide number), per individual animals in the vehicle (green), semaglutide (red) and control (blue) groups.

**Supplementary Figure 10. Tuncia media area versus depth (slide number) for the control study (constrictive cuff)**


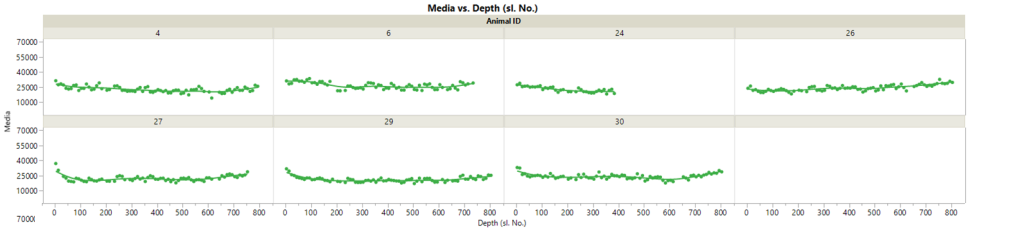


Tunica media area versus depth of the LCCA (slide number), per individual animals.

**Supplementary Figure 11. Coagulative markers in animals from Study B**

**
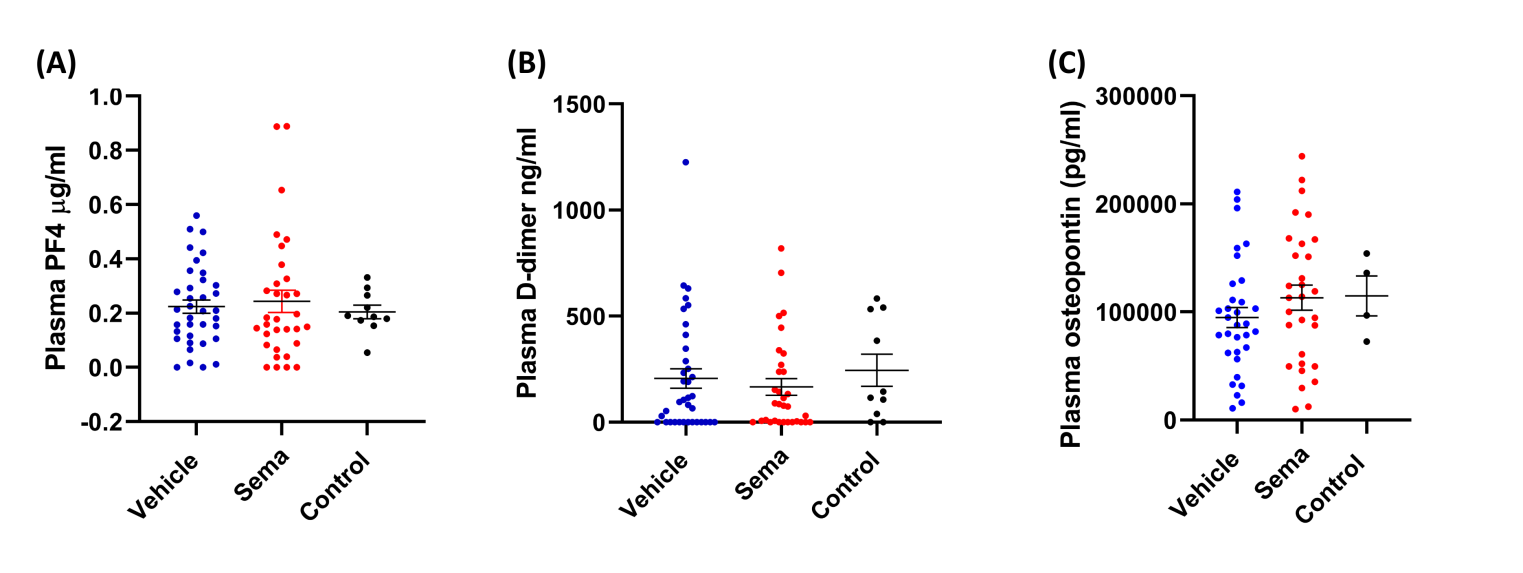
**

(A) Platelet factor 4 (PF4) levels in plasma in Study B. (B) D-dimer levels in plasma in Study B. (C) Osteopontin levels in plasma in Study B.

**Supplementary Figure 12. Spatial transcriptomics performed on human coronary vessels with and without atherosclerotic plaque development**


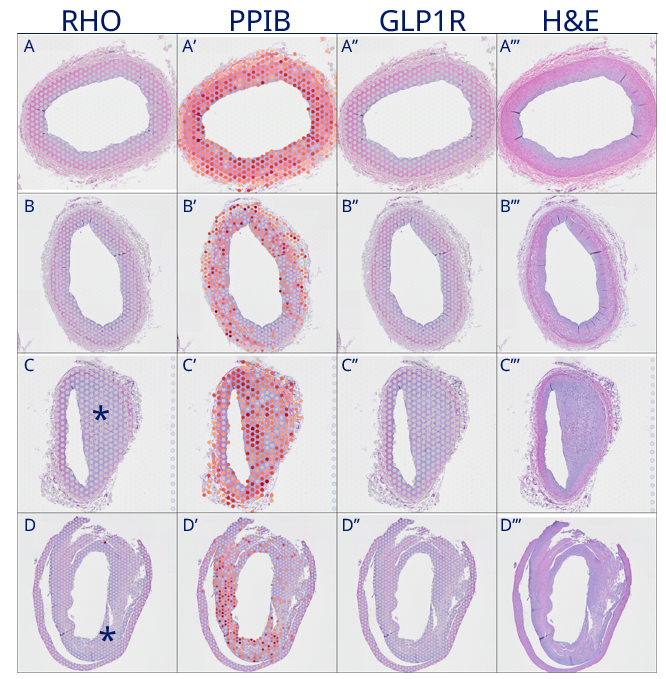


Spatial transcriptomics for RHO (negative control, A-D), PPIB (positive control, A’-D’), GLP-1R (A’’-D’’) on sections from 4 human coronary arteries (A-B without plaque, C-D with plaque (marked with asterisks)). Note absence of signal for GLP-1R similar to RHO negative control. A’’’-D’’’ is H&E staining of sections used for ST.

**Supplementary Figure 13. Immunohistochemistry and *in situ* hybridization performed on human coronary vessels with and without atherosclerotic plaque development**


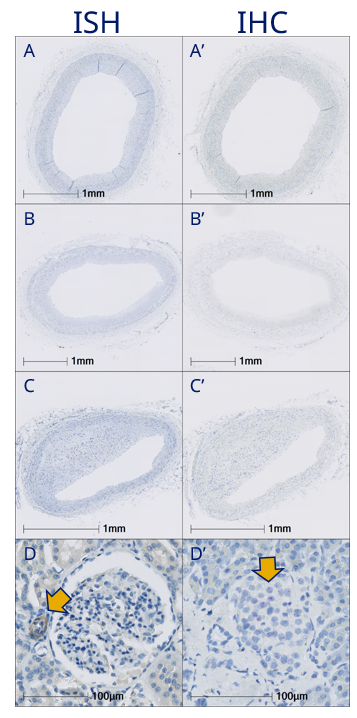


Immunohistochemistry (IHC; A-D) and *in situ* hybridization (ISH; A’-D’) for GLP-1R on near-adjacent sections from 3 human coronary arteries (A-C) also used for ST (A-C in Supplementary Figure 12). Note absence of signal for GLP-1R using both methods. D is positive control for IHC (monkey kidney, brown staining in afferent arteriole (arrow)) and D’ is positive control for ISH (human pancreas, red dots in islet (arrow)).

**Supplementary Figure 14. Human coronary artery smooth muscle cell proliferation.**

**
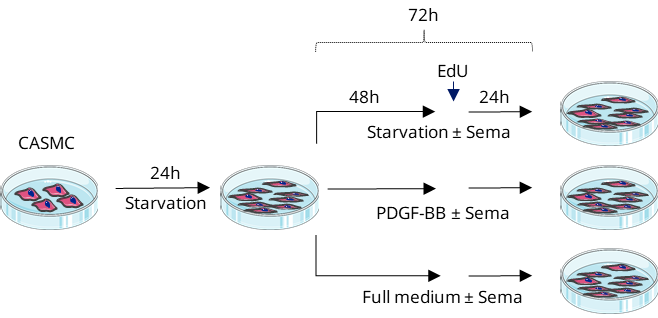
**

**A**

**C**

**B**


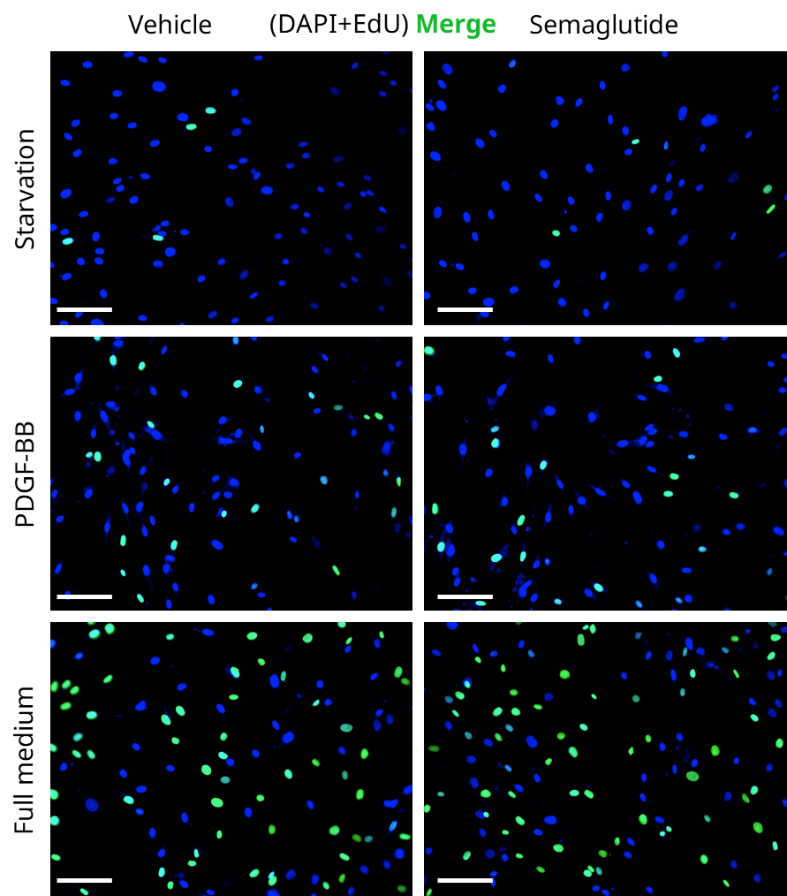

(A) Experimental timeline. Starved human coronary artery smooth muscle cells (hCASMCs) were treated with vehicle or 1µM Semaglutide (Sema) in either starvation, PDGF-BB (20ng/mL in starvation) or full medium for 72h. 24h prior to the experiment completion, EdU was added (B) Representative images of EdU incorporation with EdU (Green) and DAPI (Blue) cells. (C) % EdU^+^ cells following vehicle or sema treatment. Scale bar =100 μm. More than 8000 cells were analyzed/condition per donor. Data shown are means±SEM, *n* = 3 independent donors in quadruplicates, ns-non significant (*p* > 0.05), one-way ANOVA, with Dunnett’s correction test.

**Supplementary table 1**

| **Staining / Marker** | **Detailed information** |
| --- | --- |
| Pentachrome | Movat Pentachrome Stain Kit, Abcam, catalog number ab245884 |
| CD31 | Monoclonal rabbit anti-mouse CD31 antibody, clone D8V9E, Cell Signaling Technology, catalog number 77699. Dilution applied to slides: 1:100 |
| Mac-2 | Monoclonal rat anti-mouse/human Mac-2 IgG2 antibody, clone M3/38, Biolegend, catalog number 125401. Dilution applied to slides: 1:2000 |
| Myh11 mRNA | RNAscope® 2.5 VS Probe-Mm-Myh11, catalog number 316109, Advanced Cell Diagnostics |
| Osteopontin (SPP1) mRNA | RNAscope® 2.5 VS Probe-Mm-Spp1-C2, catalog number 435199-C2 , Advanced Cell Diagnostics |
| PPIB mRNA | RNAscope® 2.5 VS Probe-Mm-Ppib-C2, catalog number 313919-C2, Advanced Cell Diagnostics |
| DapB mRNA | RNAscope® 2.5 VS Negative Control probe DapB-C2, catalog number 312039-C2, Advanced Cell Diagnostics |

**References**

[1] G. Franck, T. Mawson, G. Sausen, M. Salinas, G.S. Masson, A. Cole, M. Beltrami-Moreira, Y. Chatzizisis, T. Quillard, Y. Tesmenitsky, E. Shvartz, G.K. Sukhova, F.K. Swirski, M. Nahrendorf, E. Aikawa, K.J. Croce, P. Libby, Flow Perturbation Mediates Neutrophil Recruitment and Potentiates Endothelial Injury via TLR2 in Mice, Circ. Res. 121 (2017) 31–42. https://doi.org/10.1161/CIRCRESAHA.117.310694.

[2] K.S.P. Abelson, K.R. Jacobsen, R. Sundbom, O. Kalliokoski, J. Hau, Voluntary ingestion of nut paste for administration of buprenorphine in rats and mice, Lab. Anim. 46 (2012) 349–351. https://doi.org/10.1258/la.2012.012028.

[3] G. Franck, G. Even, A. Gautier, M. Salinas, A. Loste, E. Procopio, A.T. Gaston, M. Morvan, S. Dupont, C. Deschildre, S. Berissi, J. Laschet, P. Nataf, A. Nicoletti, J.B. Michel, G. Caligiuri, Haemodynamic stress-induced breaches of the arterial intima trigger inflammation and drive atherogenesis, Eur. Heart J. 40 (2019) 928–937. https://doi.org/10.1093/eurheartj/ehy822.

[4] P. Carmeliet, L. Moons, J. Stassen, M. De Moi, A. Bouché, J.J. vand den Orrd, M. Kockx, D. Collen, Vascular Wound Healing and Neointima Formation Induced by Perivascular Electric Injury in Mice, Am. J. Pathol. 150 (1997) 761–776.

[5] G. Franck, T. Mawson, G. Sausen, M. Salinas, G.S. Masson, A. Cole, M. Beltrami-Moreira, Y. Chatzizisis, T. Quillard, Y. Tesmenitsky, E. Shvartz, G.K. Sukhova, F.K. Swirski, M. Nahrendorf, E. Aikawa, K.J. Croce, P. Libby, Flow Perturbation Mediates Neutrophil Recruitment and Potentiates Endothelial Injury via TLR2 in Mice: Implications for Superficial Erosion, Circ. Res. 121 (2017) 31–42. https://doi.org/10.1161/CIRCRESAHA.117.310694.
